# Association of psychiatric illness with acute outcomes following emergency general surgery

**DOI:** 10.1016/j.sopen.2025.12.001

**Published:** 2025-12-08

**Authors:** Giselle Porter, Sara Sakowitz, Syed Shaheer Ali, Troy Coaston, Konmal Ali, Amulya Vadlakonda, Zihan Gao, Peyman Benharash

**Affiliations:** a Center for Advanced Surgical & Interventional Technology, University of California – Los Angeles, Los Angeles, CA, USA; b Department of Surgery, David Geffen School of Medicine at UCLA, Los Angeles, CA, USA

**Keywords:** Emergency general surgery, Psychiatric illness, Acute care surgery, Readmissions, Nationwide readmissions database

## Abstract

**Introduction:**

Prior work has linked severe psychiatric illness (SPI) with greater postoperative mortality and complications following several elective operations. However, this relationship has not been defined in the setting of emergency general surgery (EGS).

**Methods:**

The 2016–2021 United States Nationwide Readmissions Database was used to identify all non-elective adult EGS hospitalizations performed within 48 h of admission. SPI was defined using ICD-10 codes for bipolar disorder and schizophrenia. Patients with severe psychiatric illness comprised the SPI cohort (others: Non-SPI). Multivariable linear and logistic regression models were developed to evaluate the independent association of severe psychiatric illness with in-hospital mortality, perioperative complications, and costs.

**Results:**

Of 2,124,284 EGS patients, 52,130 (2.5 %) were categorized as SPI. On adjusted analysis, SPI was associated with greater odds of in-hospital mortality (Adjusted Odds Ratio [AOR] 1.21; 95 % Confidence Interval [CI]:1.11–1.31), hospitalization costs (+$2304; 95 %CI: +1950, +2658), as well as a 3-fold increase in relative risk of non-home discharge (AOR 3.18, 95 % CI: 3.05–3.31).

**Conclusions:**

Among EGS patients, severe psychiatric illness was linked with inferior clinical and financial outcomes. Improved psychiatric screening and care may allow for early intervention and targeted postoperative care, potentially mitigating complications and costs for these vulnerable patients.

## Graphical abstract

**Figure:**
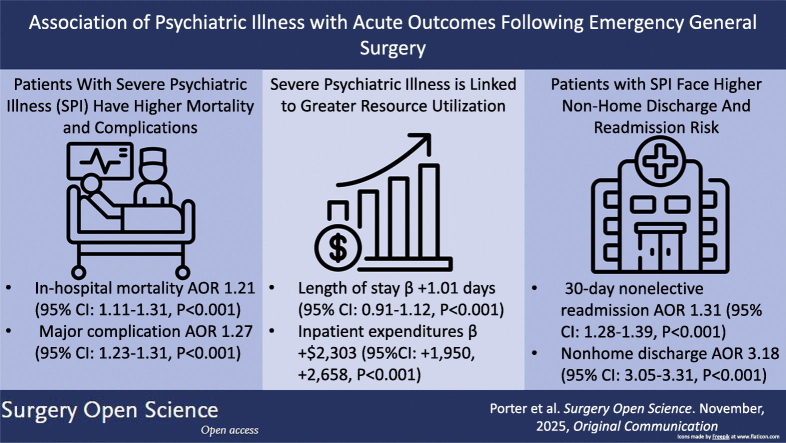

## Introduction

Severe psychiatric illness (SPI), defined as bipolar, schizophrenia, and schizophreniform disorders, are present in up to 30 % of patients undergoing elective general surgery [1]. Indeed, SPI has been linked to increased mortality and perioperative complications after elective procedures spanning oncologic, geriatric, and cardiac specialties [[2], [3], [4]]. The mechanisms underlying such adverse events are likely multifactorial, and may reflect the complex needs for social support and care coordination in this population [5].

Contemporary literature has predominantly focused on physical comorbidities for risk stratification and optimization in, overlooking the significant impact of mental health on treatment outcomes. However, within elective procedures, this particularly vulnerable cohort of patients face a higher incidence of perioperative complications, with nearly half requiring extended hospitalization [2,4,6]. Consequently, SPI patients are frequently burdened with a greater likelihood of readmission and increased out-of-pocket costs [1,2,7]. Yet, such outcomes remain unexplored in emergency general surgery. Indeed, the non-elective setting poses unique perioperative and economic challenges given the inherent case complexity of EGS and inadequate time for preoperative optimization. The historical neglect of mental health is widely acknowledged to have far-reaching ramifications, and merits study among patients requiring EGS.

In the present study, we characterized the association of severe psychiatric illness with acute outcomes of emergency general surgery procedures in a national cohort. We hypothesized SPI to be linked with a higher risk of in-hospital mortality and complications as well as markers of resource use and nonelective readmissions within 30 days of index discharge.

## Methods

### Data source and study population

The 2016–2021 Nationwide Readmissions Database (NRD) was used to identify all non-elective adult (≥18 years) hospitalizations entailing EGS (appendectomy, laparotomy, large and small bowel resection, lysis of adhesions, perforated ulcer repair) within two days of admission, using previously validated *International Classification of Diseases, Tenth Revision* (ICD-10) codes [8,9]. The NRD is the largest all-payer inpatient readmissions database in the US and provides accurate estimates for ~60 % of all annual hospitalizations. Through utilization of unique patient identifiers, the NRD permits the tracking of readmissions within each state and calendar year.

### Severe psychiatric illness

**Fig. 1:**
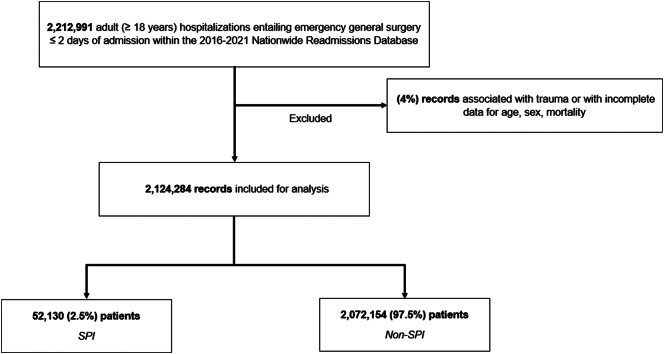
Study CONSORT diagram of survey-weighted estimates. *SPI, Severe Psychiatric Illness.*

Severe psychiatric illness (SPI) was defined using previously validated ICD-10 codes for bipolar and schizophrenia disorders [4,7,10]. Patients with at least one diagnosis of bipolar disorder or schizophrenia were classified as the *SPI* cohort (others: *Non-SPI*). Records entailing traumatic injury at index hospitalization or missing key data were excluded from further analysis (4 %) (Fig. 1).

### Variable definitions and study outcomes

Patient and hospital characteristics, including age, sex, insurance, and hospital teaching status, were defined using the NRD data dictionary [11]. Patient burden of chronic conditions was quantified using the van Walraven modification of the Elixhauser Comorbidity Index [12]. We used previously reported ICD*-*10 codes to ascertain the presence of comorbidities and incidence of perioperative complications [13]. Major adverse events were classified as cardiac (cardiac arrest, ventricular tachycardia, ventricular fibrillation, cardiac tamponade, myocardial infarction), thromboembolic (deep vein thrombosis, pulmonary embolism), respiratory (pneumonia, postprocedural pneumothorax, ARDS, respiratory failure, prolonged ventilation), infectious (urinary tract infection, unspecified postoperative infection, systemic inflammatory response with or without organ dysfunction, sepsis, septicemia, bacteremia, post-operative seroma, wound disruption, *Clostridium difficile* infection, peritoneal abscess, cellulitis, mediastinitis, surgical site infection, colostomy infection), intraoperative (accidental puncture, hemorrhage), stroke, and acute kidney injury. Non-home discharge was considered as disposition to a short-term hospital or skilled nursing facility. To calculate hospitalization costs, unique hospital cost-to-charge ratios were applied to overall charges and adjusted to the 2020 Personal Healthcare Price Index to account for inflation [14].

The primary outcome of interest was in-hospital mortality during index hospitalization. We secondarily evaluated incidence of specific perioperative complications as above, length of stay (LOS), hospitalization costs, non-home disposition, and non-elective readmissions within 30 days of index discharge.

### Statistical analysis

Categorical variables are reported as proportions (%), while continuous variables are presented as medians with interquartile range (IQR). Bivariate comparisons were performed using the Adjusted Wald, Mann-Whitney U, and Pearson's X^2^ tests, as appropriate. Cuzick's nonparametric test (nptrend) was utilized to evaluate the significance of temporal trends [15]. Multivariable regression models were subsequently employed to evaluate the independent associations between SPI and outcomes of interest. Model performance was evaluated using receiver-operating-characteristics (C-statistic) or coefficient of determination (R^2^), as appropriate. Model estimates are reported as adjusted odds ratios (AOR), or beta coefficients (β), both with 95 % confidence intervals (95 % CI). Statistical significance was considered at α of 0.05. All statistical analyses were performed using Stata 18.0 software (StataCorp, College Station, TX). Due to the deidentified nature of the NRD, this study was deemed exempt from full review by the Institutional Review Board at the University of California, Los Angeles.

## Results

Of an estimated 2,124,284 emergency general surgery (EGS) patients meeting inclusion criteria, 52,130 (2.5 %) were classified as *SPI* (others: *Non-SPI*)*.* Compared to others, *SPI* patients were younger, (53 [40–63] vs 59 years [43–72], *p* < 0.001), more commonly female (63.3 vs 58.9 %, p < 0.001), of a greater comorbidity burden (3 [2–4] vs 2 [1–4], *p* < 0.001), and more commonly classified in the lowest income quartile (34.1 vs 27.7 %, *P* < 0.001). Additionally, *SPI* had a higher prevalence of chronic lung disease (24.3 vs 13.6 %, *p* < 0.001), liver disease (9.1 vs 7.8 %, *p* < 0.001), obesity (28 vs 23.4 %, p < 0.001) and smoking (51.8 vs 33 %, *p* < 0.001) (Table 1). While cholecystectomy remained the most common EGS operation in both groups (47.1 % vs 52.3 %; p < 0.001), *SPI* more frequently received lysis of adhesions (19.2 vs 16.3 %; p < 0.001) compared to *Non-SPI.*

**Table 1:** Demographic and hospital characteristics.⁎

	*Non-SPI* (*n* = 2,124,184)	*SPI* (*n* = 52,130)	*P-value*
Age (years [IQR])	59 [43–72]	53 [40–63]	<0.001
Female (%)	58.9	63.2	<0.001
Elixhauser Comorbidity Index (median [IQR])	2 [1–4]	3 [2–4]	<0.001
*Income quartile (%)*			<0.001
>75 %	19.5	14.9	
51–75 %	25.0	22.2	
26–50 %	27.8	28.8	
0–25 %	27.7	34.1	
*Insurance coverage (%)*			<0.001
Private	34.6	17.1	
Medicare	41	47.3	
Medicaid	15.8	28.0	
Other Payer	8.6	7.6	
*Comorbidities (%)*			
Hypertension	45.8	42.5	<0.001
Cardiac Arrhythmia	13.9	10.7	<0.001
Congestive Heart Failure	7.3	6.9	0.014
Chronic Kidney Disease	1.6	1.3	<0.001
Diabetes	17.7	20.4	<0.001
Chronic Lung Disease	13.6	24.4	<0.001
Neurologic Disorder	5	13.2	<0.001
Liver Disease	4.3	4.8	<0.001
Peripheral Vascular Disease	5.7	5.5	0.15
Pulmonary Circulatory Disease	1.6	1.3	<0.001
Smoker	33.0	51.8	<0.001
Obesity	23.4	28	<0.001
*Hospital Characteristics*			<0.001
Non-Metropolitan Non-Teaching	8.8	9.4	
Metropolitan Non-Teaching	24.4	23	
Metropolitan Teaching	66.8	67.6	

Reported as proportions unless otherwise noted.

⁎ IQR, interquartile range; SPI, Severe Psychiatric Illness.

On unadjusted analysis, *SPI* patients experienced statistically different, but clinically comparable rates of in-hospital mortality (2.8 vs 2.7 %, *p* < 0.05) and increased rates of any perioperative complication (36.8 vs 30 %, *p* < 0.001), including respiratory (8.8 vs 5.8 %, p < 0.001), infectious (26.8 vs 20.4 %, *p* < 0.001), and renal (16.6 vs 13.4 %, p < 0.001) sequelae. Notably, *SPI* more often underwent non-home disposition (18 vs 9.5 %, p < 0.001) and were more often readmitted within 30 days of index discharge (12.4 vs 8.2 %, p < 0.001) (Table 2).

**Table 2:** Unadjusted and adjusted outcomes, stratified by presence of severe psychiatric illness of patients undergoing emergency general surgery from 2016 to 2021.⁎

	Unadjusted			Adjusted		
	*Non-SPI*	*SPI*	*P*	*SPI*	*95* *% CI*	*P*
Clinical outcomes						
In-Hospital mortality (%)	2.7	2.9	0.05	1.21	1.11–1.31	<0.001
Complications						
Stroke	0.13	0.14	0.66	1.17	0.83–1.65	0.38
Thromboembolic	0.6	0.7	0.21	0.84	0.71–0.99	0.04
Intraoperative	1.8	1.8	0.77	1.01	0.92–1.12	0.81
Cardiac	2.1	2.1	0.84	1.24	1.13–1.37	<0.001
Blood Transfusion	5.5	6.1	<0.001	0.96	0.91–1.02	0.16
Respiratory	5.8	8.8	<0.001	1.48	1.41–1.56	<0.001
Renal	13.4	16.6	<0.001	1.28	1.23–1.34	<0.001
Infectious	20.4	26.8	<0.001	1.36	1.32–1.40	<0.001
Any Complication	30	36.8	<0.001	1.27	1.23–1.31	<0.001
Resource utilization						
Non-home discharge	9.5	18	<0.001	3.18	3.05–3.31	<0.001
LOS (days) [IQR]	4 [2–7]	4 [2–9]	<0.001	1.01	0.91–1.12	<0.001
Costs (USD $1000) [IQR]	$15,588 [10,917-24,026]	$17,462 [11,796-28,758]	<0.001	$2304	1950-2658	<0.001
30-Day non-elective readmission	8.2	12.4	<0.001	1.33	1.28–1.39	<0.001

Outcomes reported as proportions or as Adjusted Odds Ratio (AOR) with 95 % confidence intervals (95 % CI). Reference: Non-SPI.

⁎ IQR, interquartile range; USD, United States dollar; SPI, Severe Psychiatric Illness.

**Fig. 2:**
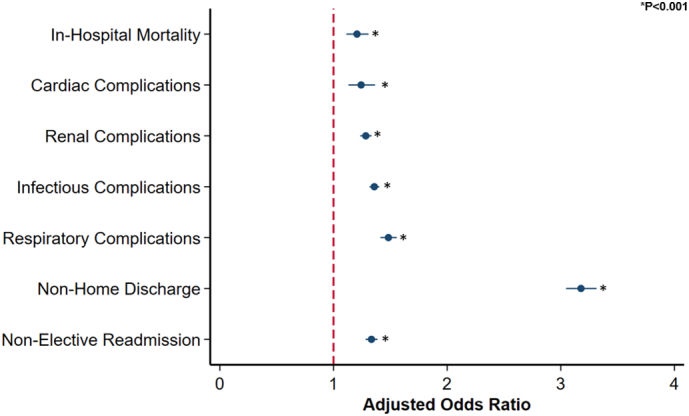
Association of severe psychiatric illness with perioperative outcomes in emergency general surgery. *Indicates statistical significance, P *<* *0.001. Error bars represent 95* *% confidence intervals. Ref: Non-SPI.*

**Fig. 3:**
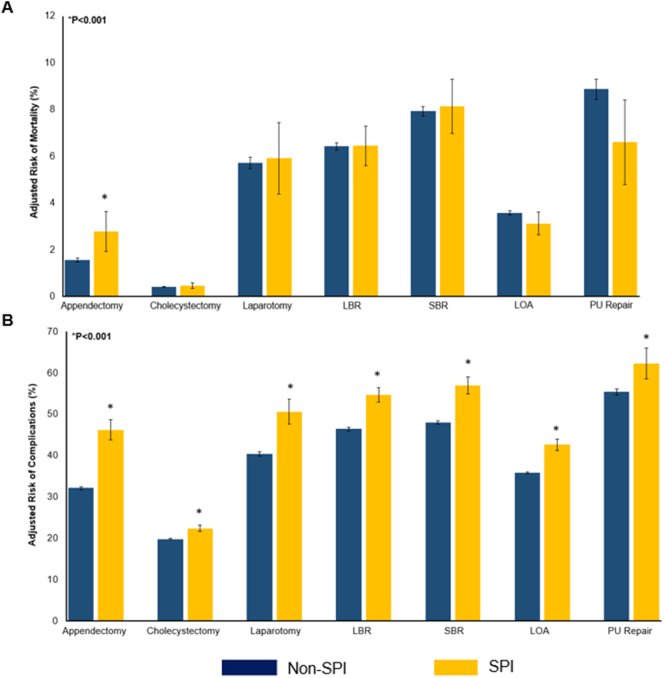
Adjusted risk of in-hospital A. Mortality and B. Complications, stratified by presence of psychiatric illness. *Indicates statistical significance, P *<* *0.001. Error bars represent 95* *% confidence intervals. SPI, Severe Psychiatric Illness; LBR, large bowel resection; SBR, small bowel resection; LOA, lysis of adhesions; PU Repair, peptic ulcer repair.*

Following comprehensive risk adjustment, SPI was associated with increased odds of in-hospital mortality (AOR 1.21 95 % CI:1.11–1.31). Additionally, SPI was linked with greater odds of cardiac (AOR 1.24, 95 % CI: 1.13–1.37), respiratory (AOR 1.48, 95 % CI: 1.41–1.56), infectious (AOR 1.36, 95 % CI: 1.32–1.40), and renal complications (AOR 1.28, 95 % CI: 1.23–1.34) (Fig. 2). Moreover, SPI demonstrated increased risk-adjusted probability of perioperative complications across all procedure types (Fig. 3).

**Fig. 4:**
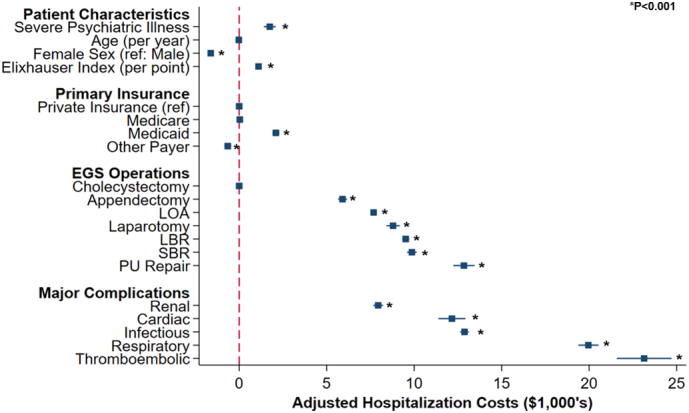
Factors associated with differences in adjusted hospitalization costs. *Indicates statistical significance, P* *<* *0.001. Error bars represent 95* *% confidence intervals. LBR, large bowel resection; SBR, small bowel resection; LOA, lysis of adhesions; PU Repair, peptic ulcer repair.*

**Fig. 5:**
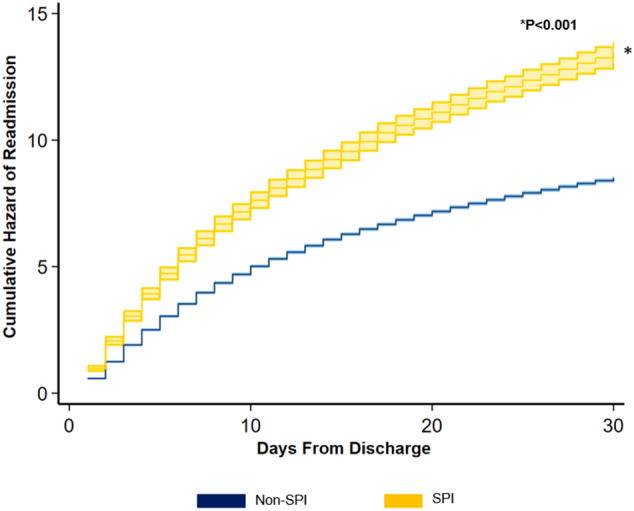
Cumulative risk of non-elective readmission within 30 days. (Reference: *Non-SPI*). *Indicates statistical significance, P* *<* *0.001. Error bars represent 95* *% confidence intervals. SPI, Severe Psychiatric Illness.*

Evaluating resource utilization, SPI was linked to greater length of stay (β +1.01 days; 95 % CI: 0.91–1.12), inpatient expenditures (+$2303; 95 %CI: +1950, +2658), and odds of non-home discharge (AOR 3.18,95 % CI: 3.05–3.31). The presence of severe psychiatric illness, Elixhauser index, and development of major perioperative complications including renal (β + $7900; 95 % CI: +$7700 - $8200, *P* < 0.001), cardiac (β + $12,300; 95 % CI: +$11,400 - $12,900, P < 0.001), infectious (β + $12,900; 95 % CI: +$12,600 - $13,100 P < 0.001), respiratory (β + $20,000; 95 % CI: +$19,400 - $20,500, P < 0.001), and thromboembolic complications (β + $23,100; 95 % CI: +$21,600 - $24,700, P < 0.001), were independently linked with increased costs. Furthermore, all EGS operation types were associated with increased hospitalization costs. Conversely, female sex and other payer were associated with decreased risk-adjusted costs. Additionally, *SPI* patients faced significantly increased risk of non-elective readmission within 30 days of index discharge (Table 2) (Fig. 5) (Fig. 4).

## Discussion

A growing body of literature has identified the association of psychiatric disorders with poor clinical outcomes in the elective perioperative setting. With increasing recognition of the significance of psychiatric illness, we characterized their impact on acute outcomes following EGS at the large scale. After risk adjustment for patient- and hospital-level characteristics, severe SPI remained independently associated with greater odds of in-hospital mortality and perioperative complications. Additionally, psychiatric illness was linked to increased resource utilization including longer duration of stay, increased hospitalization costs, greater risk of non-home disposition, and higher likelihood of 30-day nonelective readmission. Indeed, while SPI has demonstrated significant association with various markers of poor prognosis across surgical specialties, we validate its significance in the context of emergency general surgery.

Across EGS operations, we identified patients with severe psychiatric illness to be at greater risk of in-hospital mortality and perioperative complications. Prior works in the elective setting have demonstrated an association between psychiatric illness and adverse outcomes in orthopedic, cardiac, and oncologic procedures [4,16,17]. A nationwide study conducted by Brown and colleagues (2021) reported a 5 % increase in the relative risk of mortality among patients with psychiatric illness [16]. In a single center study, Tyerman et al. (2021) found that although those with severe mental illness had increased comorbidity burden at baseline, a higher risk of major adverse events persisted even after comprehensive risk adjustment [17]. Our current work evaluated EGS by procedure type and identified persistent perioperative complications across EGS procedures, with appendectomy having the starkest contrast in mortality. Prior literature suggests this trend may be a result of delayed diagnosis, consequently resulting in these patients experiencing higher rates of perforation [18,19]. Additionally, patients with severe mental illness often require prolonged hospital stays which expose them to greater risk of nosocomial complications [20,21]. Irrespective of the stark increase in perioperative complications observed among those with psychiatric illness, these disparities are likely underestimated. With an insufficient mental health infrastructure, disparities in availability of mental health resources, and the persistent societal stigma surrounding mental health, psychiatric illness is vastly underdiagnosed. Therefore, the association of these diagnoses with surgical outcomes may not be accurately understood.

The present study demonstrated severe psychiatric illness to be independently associated with increases across all markers of resource utilization, including length of stay, hospitalization costs, non-home discharge, and non-elective readmission. In a single center study conducted by McBride et al., psychiatric illness was associated with longer hospital stays and an increment of over $10,000 in hospitalization costs [22]. Furthermore, increased rates of respiratory, infectious, renal, and thromboembolic complications seen among this cohort of patients is likely responsible for the observed rise in hospitalization costs and non-elective readmission. Indeed, there is an obvious need for care pathways capable of mitigating perioperative risk in the post-acute setting, ultimately decreasing length of stay and overall costs. Implementing interdisciplinary care teams that prioritize psychiatric care may provide actionable means to reduce resource utilization. Finally, we observed an over three-fold increase in non-home disposition among those with severe psychiatric illness. Rapisarda and colleagues (2020) concluded the complex care needs of patients with psychiatric illness require management at long-term care facilities, representing an obstacle to the efficient use of acute care [23]. Interdisciplinary care emphasizing psychiatric management along with specialized discharge planning may enhance value-based care among this cohort of patients [23].

With a lack of standardized infrastructure to manage psychiatric illness in the peri-acute setting, our study joins a growing body of literature informing modalities for improvement. Association of psychiatric illness with inferior perioperative outcomes is likely multi-factorial and include lifestyle predispositions, non-adherence to postoperative care, and inadequate healthcare infrastructure for psychiatric care. Chronic inflammation may be a potential mediator between severe psychiatric illness and adverse surgical outcomes. Such conditions have been associated with elevated inflammation which may contribute to impaired wound healing, infection, and other perioperative complications. While the exact mechanisms driving poor outcomes is unclear, this disparity represents a major area for optimization. Recent work suggests developing more robust mental healthcare protocols could mitigate inferior outcomes [7]. Broader screening and treatment for psychiatric needs at the earliest feasible opportunity in the acute setting through use of interdisciplinary teams has shown promise in improving outcomes post-surgically [24,25]. Such studies emphasized cost-effective utilization of psychiatric screening, compassionate education, and early psychiatric intervention as critical means of enhancing efficient use of acute care. Furthermore, previous studies have validated the use of prehabilitation in the setting of major surgical intervention [26,27]. Though this is not realistic in the acute-care setting, rehabilitation in the post-acute setting may be feasible. Indeed, a holistic care plan emphasizing physical and mental health may ameliorate resource utilization and poor post-operative outcomes for these patients.

The present study has several important limitations. Because the NRD is an administrative database, we do not have access to granular laboratory or imaging data. The NRD also relies on accurate ICD coding which can vary greatly based on center and physician billing practices. Moreover, we are unable to track patient survival beyond hospitalization and therefore are unable to make conclusions surrounding the impact of severe psychiatric illness on long-term outcomes. However, the present study employed robust statistical methods on a large, nationally representative cohort to evaluate the association between severe psychiatric illness and outcomes following emergency general surgery.

In sum, our analysis adds to the growing work evaluating psychiatric illness as a crucial comorbidity for surgical care optimization. Indeed, despite the underestimation of psychiatric illness in the acute setting, the perioperative disparities are still apparent. Severe psychiatric illness is disproportionately associated with greater risk of perioperative complications, mortality during index hospitalization, increased length of stay, and hospitalization costs. Our analyses further corroborate the value of incorporating mental health in risk stratification in the post-acute setting in an effort to optimize post-discharge planning. Considering the strong association between psychiatric illness and major morbidity, it is essential to invest in mental health as an integral component of interdisciplinary care paradigms in the acute setting.

## Funding sources statement

The authors declare that no external funding was received for this work.

## CRediT authorship contribution statement

**Giselle Porter:** Writing – review & editing, Writing – original draft, Visualization, Validation, Methodology, Investigation, Formal analysis, Data curation, Conceptualization. **Sara Sakowitz:** Writing – review & editing, Validation, Methodology, Conceptualization. **Syed Shaheer Ali:** Writing – review & editing, Visualization, Validation. **Troy Coaston:** Writing – review & editing, Validation. **Konmal Ali:** Writing – review & editing, Investigation. **Amulya Vadlakonda:** Writing – review & editing, Investigation. **Zihan Gao:** Writing – review & editing, Investigation. **Peyman Benharash:** Writing – review & editing, Validation, Supervision, Resources, Project administration, Investigation.

## Ethical approval statement

This study did not require ethical approval as no human subjects were directly involved, and only publicly available, de-identified data were used.

## Declaration of competing interest

The authors declare that no external funding was received for this work. Dr. Peyman Benharash discloses being a proctor for Atricure; however, this relationship is unrelated to the present study.
